# Supplementary material for: The Holocene retreat dynamics and stability of Petermann Glacier in northwest Greenland
Source: Nat Commun. 2018 May 29;9:2104. doi: 10.1038/s41467-018-04573-2 (PMC5974188; doi:10.1038/s41467-018-04573-2)
Supplement: Supplementary file 1 — Supplementary Information [file 41467_2018_4573_MOESM1_ESM.pdf]

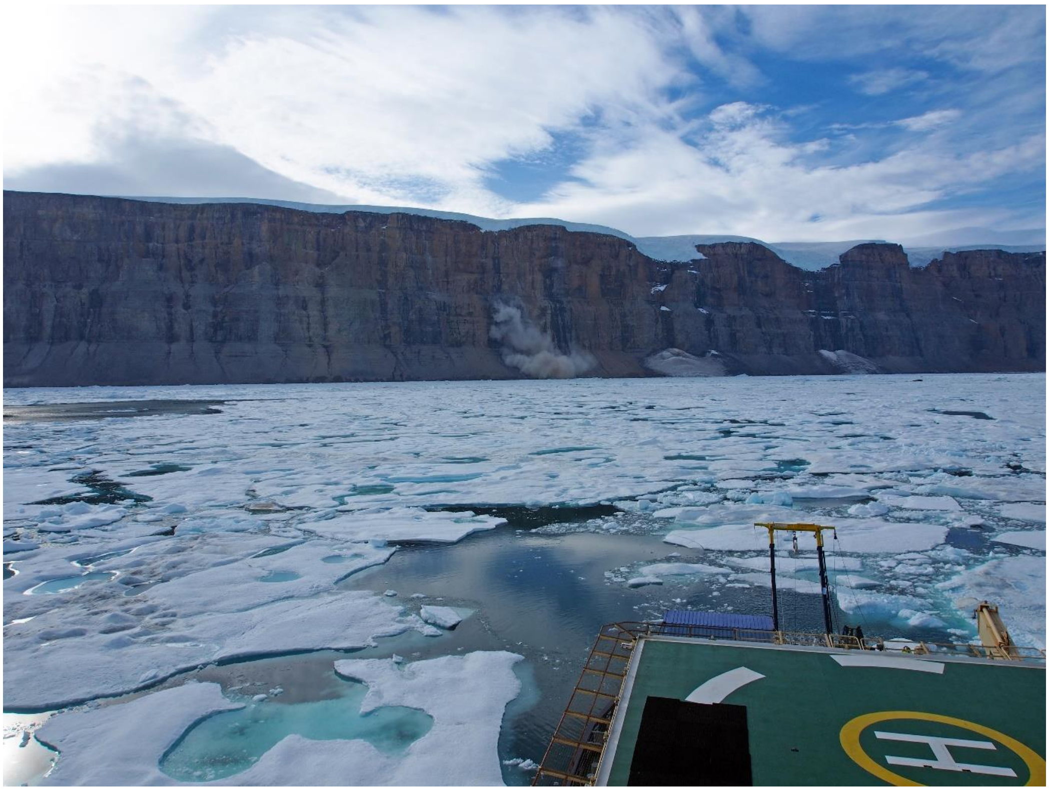

Supplementary Figure 1: Mass wasting at the western wall of Petermann Fjord south of a hanging glacier at 61°51.1’W, 80°57.5’N. The photo is taken from Swedish icebreaker (IB) *Oden*. Photo: Martin Jakobsson.

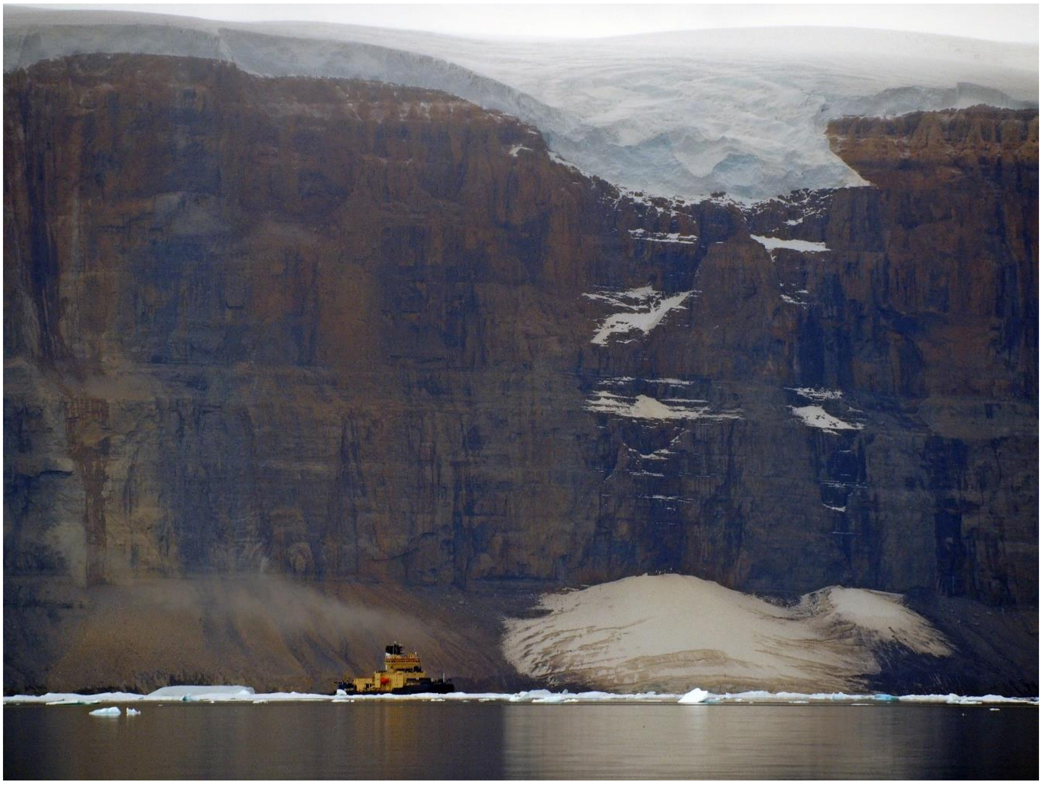

Supplementary Figure 2: IB *Oden* (108 m long) south of the hanging glacier closest to the mass wasting event shown in Photo 1. Photo: Martin Jakobsson.

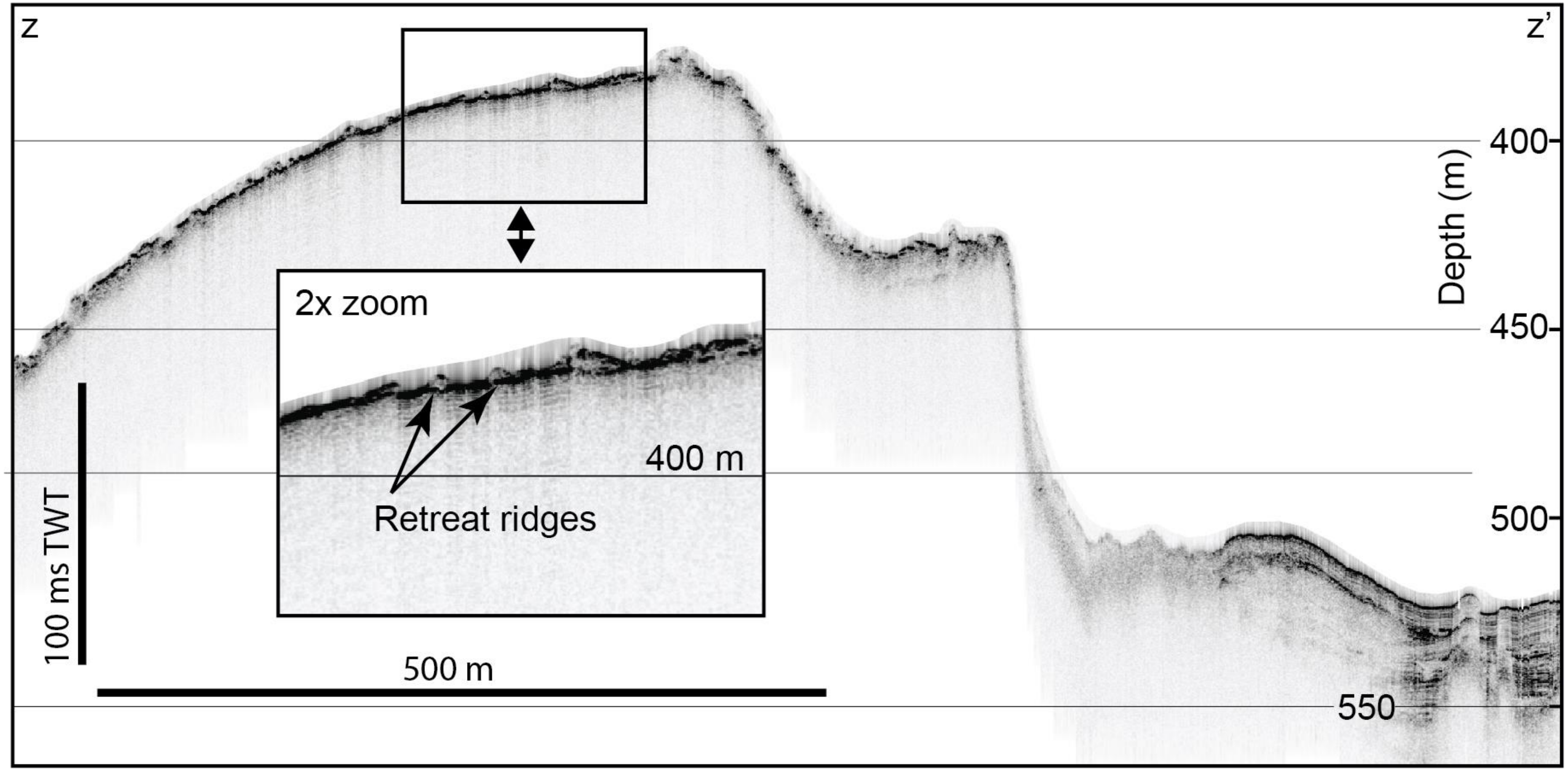

Supplementary Figure 3: Chirp sonar sub-bottom profile from south (Z) to north (Z') across the GZW at the Petermann Fjord mouth. The location of the profile is shown in Figure 2c. The small retreat ridges on the retrograded slope of the GZW's distal side are clearly visible in the enlarged inset. Vertical exaggeration is 2.6.

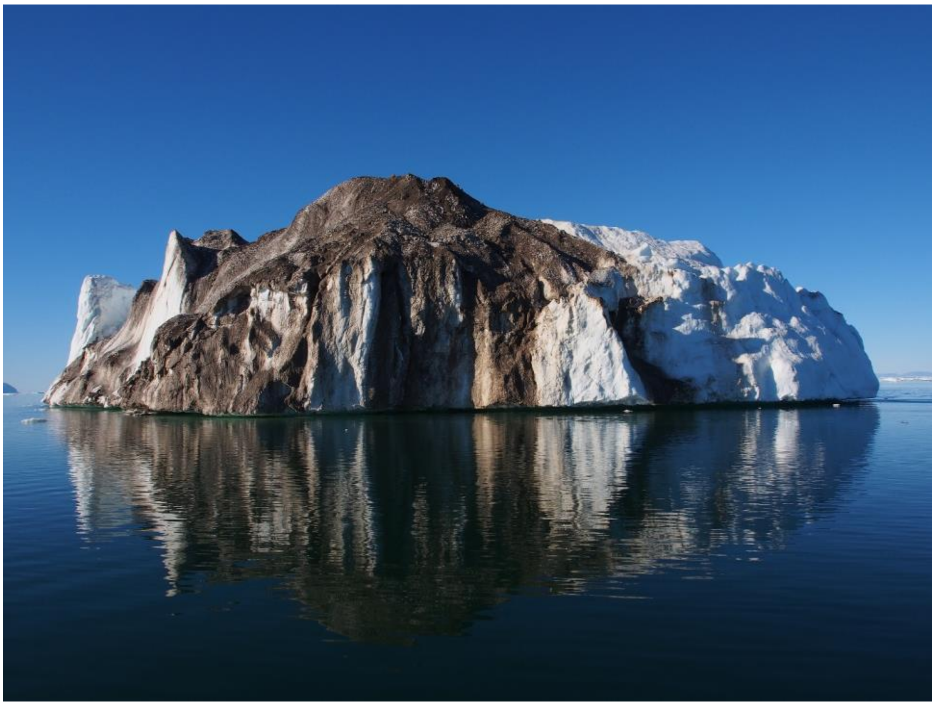

Supplementary Figure 4: One of the smaller icebergs originating from the calving of the Petermann Ice Tongue that occurred 2015-08-02. The iceberg is grounded in water depths between about 67 and 70 m and the generated ploughmark was mapped with RV *Skidbladner* (Figure 6a). Photo: Martin Jakobsson.

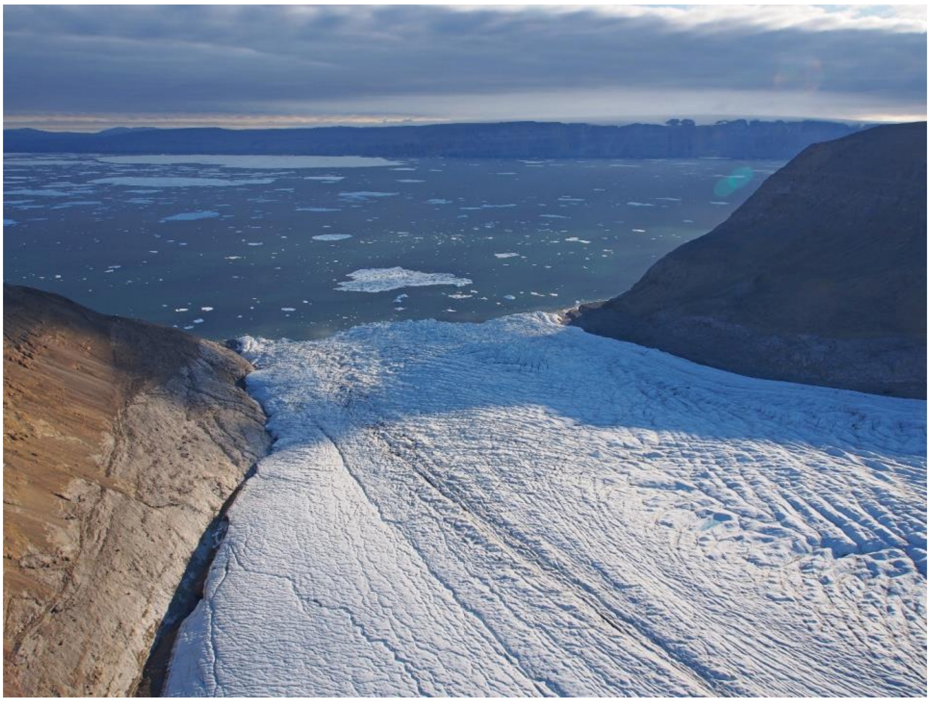

Supplementary Figure 5: Skidbladner glacier draining into the north-western side of Petermann Fjord.   Iceberg plough ridges are mapped in the seafloor along the margin of the glacier (Figure 6b).

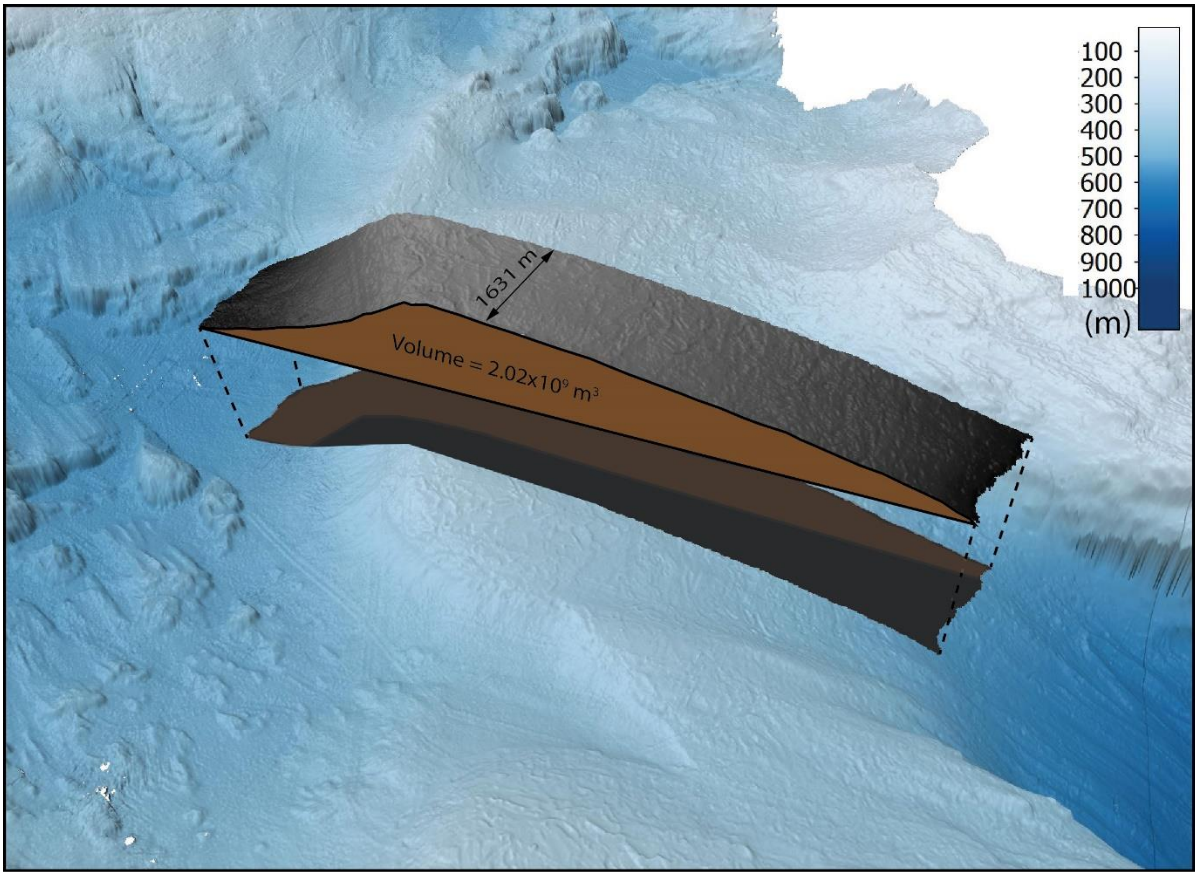

Supplementary Figure 6: Illustration showing how the volume of the GZW at the Petermann Fjord mouth was calculated in order to be used for an estimation of its formation time using different sediment flux rates (Supplementary Figure 7). A 1631 m wide slice was cut out of the wedge by inserting a plane at the foot of the GZW facing Hall Basin. The foot is here located at a present water depth of 580 m, implying that the plane was inserted at this depth to form the base of the slice. The volume of the slice was estimated to ~2.02×10^6 m^3. An average volume for 1 m wide sub-slices was derived by dividing the entire volume by 1631 m. This average volume for a 1 m wide sub-slice was used in the calculation of the GZW formation time shown in Supplementary Figure 7.

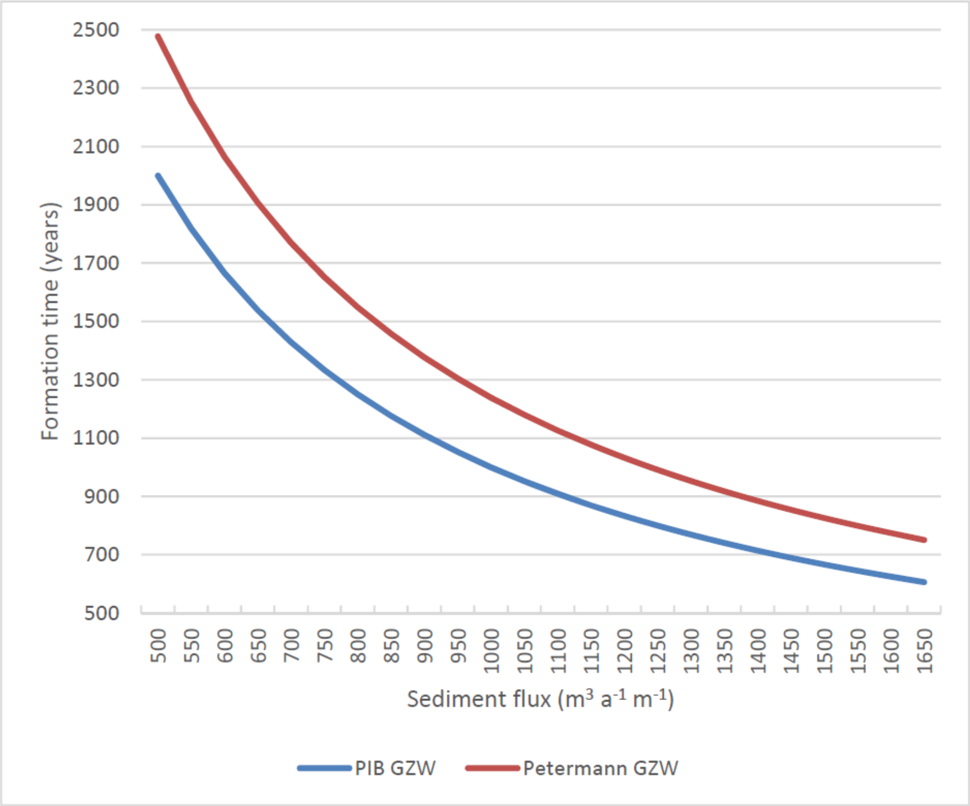

Supplementary Figure 7: Estimated formation time in years of the Petermann GZW using sediment flux rates between 500 and 1650 m^3 a^{-1} m^{-1}. These flux rates were used in a study by Jakobsson et al. [1] to estimate the formation time of the largest GZW (GZW5) mapped in the Pine Island Bay Trough, west Antarctica, and are also in line with flux rates estimated for West Greenland outlet glaciers by Hogan et al. [2]. The calculated formation time for the Pine Island Bay GZW5 is show as a comparison.

References

1 Jakobsson, M. *et al.* Ice sheet retreat dynamics inferred from glacial morphology of the central Pine Island Bay Trough, West Antarctica. *Quaternary Science Reviews* **38**, 1-10, doi:10.1016/j.quascirev.2011.12.017 (2012).

2 Hogan, K. A., Dowdeswell, J. A. & ÓCofaigh, C. Glacimarine sedimentary processes and depositional environments in an embayment fed by West Greenland ice streams. *Marine Geology* **311-314**, 1-16, doi:10.1016/j.margeo.2012.04.006 (2012).
